# The “one-step” *Bean pod mottle virus* (BPMV)-derived vector is a functional genomics tool for efficient overexpression of heterologous protein, virus-induced gene silencing and genetic mapping of BPMV R-gene in common bean (*Phaseolus vulgaris* L.)

**DOI:** 10.1186/s12870-014-0232-4

**Published:** 2014-08-29

**Authors:** Stéphanie Pflieger, Sophie Blanchet, Chouaib Meziadi, Manon MS Richard, Vincent Thareau, Fanny Mary, Céline Mazoyer, Valérie Geffroy

**Affiliations:** CNRS, Institut de Biologie des Plantes, UMR 8618, Université Paris Sud, Saclay Plant Sciences (SPS), 91405 Orsay, France; Univ Paris Diderot, Sorbonne Paris Cité, 75205 Paris, France; INRA, Unité Mixte de Recherche de Génétique Végétale, Université Paris Sud, IDEEV FR3284, Ferme du Moulon, 91190 Gif-sur-Yvette, France

**Keywords:** Disease resistance, Functional validation, Legume, *Phaseolus vulgaris*, RNAi, Post-transcriptional gene silencing, Soybean, Virus resistance gene

## Abstract

**Background:**

Over the last two years, considerable advances have been made in common bean (*Phaseolus vulgaris* L.) genomics, especially with the completion of the genome sequence and the availability of RNAseq data. However, as common bean is recalcitrant to stable genetic transformation, much work remains to be done for the development of functional genomics tools adapted to large-scale studies.

**Results:**

Here we report the successful implementation of an efficient viral vector system for foreign gene expression, virus-induced gene silencing (VIGS) and genetic mapping of a BPMV resistance gene in common bean, using a “one-step” BPMV vector originally developed in soybean. With the goal of developing this vector for high-throughput VIGS studies in common bean, we optimized the conditions for rub-inoculation of infectious BPMV-derived plasmids in common bean cv. Black Valentine. We then tested the susceptibility to BPMV of six cultivars, and found that only Black Valentine and JaloEEP558 were susceptible to BPMV. We used a BPMV-GFP construct to detect the spatial and temporal infection patterns of BPMV in vegetative and reproductive tissues. VIGS of the *PHYTOENE DESATURASE* (*PvPDS*) marker gene was successfully achieved with recombinant BPMV vectors carrying fragments ranging from 132 to 391 bp. Finally, we mapped a gene for resistance to BPMV (*R-BPMV*) at one end of linkage group 2, in the vicinity of a locus (*I* locus) previously shown to be involved in virus resistance.

**Conclusions:**

The “one-step” BPMV vector system therefore enables rapid and simple functional studies in common bean, and could be suitable for large-scale analyses. In the post-genomic era, these advances are timely for the common bean research community.

**Electronic supplementary material:**

The online version of this article (doi:10.1186/s12870-014-0232-4) contains supplementary material, which is available to authorized users.

## Background

Common bean (*Phaseolus vulgaris* L.) is the most important grain legume for direct human consumption in the world. This crop, a major source of dietary protein, minerals and certain vitamins, plays a significant role in human nutrition particularly in developing and underdeveloped countries [1]. The annotated common bean genome sequence was released in 2012 (http://www.phytozome.org; [2]). More recently, transcriptome analysis in common bean using high-throughput sequencing of RNA transcripts (RNA-seq) has provided data on gene expression profiles in different tissues (seeds, pods, leaves, roots, and nodules) at different development stages (http://www.phytozome.org). These recent advances have successfully resulted in the identification of a large number of genes. To assign functions to these genes and to relate these to agronomically important traits, there is now a critical need for functional genomics tools, enabling for instance reverse genetics strategies in common bean. Unfortunately, although genetic transformation of common bean is feasible, it has a low transformation efficiency, and is therefore not suitable for high-throughput functional genomics (reviewed in [3]).

Virus-induced gene silencing (VIGS) is an attractive tool for functional genomics in plants. VIGS technology relies on the ability of plant viruses to trigger a host defense mechanism related to post-transcriptional gene silencing (PTGS). VIGS requires the construction of a recombinant virus carrying a fragment of a specific endogenous gene that will be targeted by PTGS and thus be down-regulated [4]. The delivery procedure of the viral vector into plants (i.e. the primary inoculation) is a critical step in VIGS technology. It can be achieved by various techniques such as *Agrobacterium*-mediated infiltration (agro-inoculation), mechanical inoculation of *in-vitro* transcribed RNA, or biolistic delivery of infectious plasmid DNA (i.e. a DNA plasmid carrying a cDNA copy of the modified viral genome under the control of a 35S promoter) [5,6]. These delivery methods may be impractical for large-scale VIGS studies [7]. In recent years, several research groups have developed a method of inoculation using direct DNA rubbing of infectious DNA plasmids, thus precluding the need for *in vitro* transcription, biolistic delivery, or agro-inoculation procedures [8-10].

VIGS has proven to be an easy and rapid way to study the function of genes in many plant species (reviewed in [11]). To date, VIGS vectors have been developed in legumes from several plant viruses, such as the *Apple latent spherical virus* (ALSV), the *Pea early browning virus* (PEBV), and the *Bean pod mottle virus* (BPMV) (reviewed in [7]). Among these, BPMV has been the most widely used VIGS vector, and has been used mainly in soybean (*Glycine max*) to assess the function of disease resistance genes [12-14] and defense genes involved in plant-microbe interactions [15-21]. BPMV is a positive-strand RNA virus of the genus *Comovirus* from the family *Comoviridae*. BPMV was first discovered in common bean [22], but was subsequently shown to infect many other legume species such as soybean [23,24]. The genome of BPMV is bipartite, with two RNA molecules RNA1 (~6 kb) and RNA2 (~3.6 kb) that are encapsidated in separate isometric particles. RNA1 and RNA2 are expressed as polyproteins that are subsequently processed by proteinases for the synthesis of mature viral proteins. BPMV RNA1 has been shown to carry the pathogenicity component that determines foliar symptom severity [25].

In soybean, three generations of BPMV VIGS vectors have been successively developed by Zhang and Ghabrial [26] and Zhang *et al.* [10,18], with the aim of increasing the potential of BPMV as a viral vector for functional genomics [7]. In all three vectors, insertion of foreign DNA fragments for VIGS induction and/or gene expression is made in RNA2. The third-generation BPMV-derived vector, recently designed in soybean by Zhang *et al.* [10,27], presents important improvements compared to previous generations. First, cloning of foreign sequences into BPMV RNA2 is facilitated by the introduction of a *Bam*H1 restriction site after the translation stop codon of RNA2 to overcome the necessity of cloning foreign sequences in the same reading frame as the RNA2 polyprotein. Second, delivery of the BPMV vector into plants is possible via direct DNA rubbing of infectious plasmid DNA, a procedure adapted to high-throughput studies. Third, this BPMV vector is derived from the IA-Di1 isolate which induces very mild visual symptoms on infected soybean plants, thus avoiding possible interference between viral symptoms and silencing phenotypes. All these improvements make this new BPMV vector an ideal ‘one-step’ viral vector (so-called because there is no need for *in vitro* transcription, *Agrobacterium* transformation or coating to gold particles for biolistic delivery). This vector is adapted to high-throughput genomic studies and has enabled efficient, cost-effective, and simplified functional screening of genes in soybean [10].

The ‘one-step’ BPMV vector has been shown to infect common bean cv. Black Valentine [10]. Three weeks post-inoculation (wpi) of common bean plants with a BPMV-*Green Fluorescent Protein* (GFP) construct, extensive green fluorescence was visible in the upper systemic leaves and roots of infected plants [10]. To date, only one VIGS study has been reported in common bean using the first generation BPMV vector [28]. Common bean genes encoding nodulin 22 and stearoyl-acyl carrier protein desaturase were successfully silenced in cv. Black Valentine [28]. However, use of this first generation BPMV vector is limited to low-throughput VIGS studies mainly because (i) foreign sequences must be cloned in-frame into the RNA2 polyprotein and (ii) delivery into plants is achieved by viral RNAs transcribed *in vitro* from the BPMV constructs.

With the goal of adapting the ‘one-step’ BPMV vector for high-throughput VIGS studies in common bean, we first aimed to optimize the conditions for rub-inoculation of infectious BPMV-derived plasmids in common bean cv. Black Valentine. Secondly, we investigated the susceptibility to BPMV of several common bean genotypes of interest: in particular the G19833 and BAT93 genotypes for which complete genome sequences are available. We then describe the spatial and temporal infection patterns of BPMV in vegetative and reproductive tissues. In addition, gene silencing of the *PHYTOENE DESATURASE* (*PvPDS*) marker gene was tested with recombinant BPMV vectors carrying fragments of increasing size to determine the minimum insert length required for efficient *PvPDS* silencing. Finally, as the phenotype of resistance to BPMV was polymorphic between the two parental lines of a population of 77 recombinant inbred lines (RILs) used to set up the integrated linkage map of common bean [29], we aimed to investigate the segregation of resistance to BPMV in this RILs population using a BPMV-GFP construct, with the aim of mapping the corresponding gene(s).

## Results

### *Optimal conditions for direct rub-inoculation of infectious BPMV-derived plasmids in* P. vulgaris *cv. Black Valentine*

Three parameters were optimized for the delivery of infectious BPMV-derived plasmids by rub-inoculation in *P. vulgaris* cv. Black Valentine: plasmid quantity, intensity of mechanical rubbing, and number of inoculated primary leaves. We evaluated the efficiency of direct rub-inoculation of *P. vulgaris* cv. Black Valentine seedlings using two constructs: the empty BPMV VIGS vector (BPMV-0) and the vector expressing GFP (BPMV-GFP) (Table 1). The BPMV-0 and BPMV-GFP constructs were introduced into plants by rub-inoculation of primary leaves using a mix of BPMV RNA1 and RNA2 infectious plasmids. Delivery efficiency was estimated by visual inspection of viral symptoms and detection of green fluorescence under UV light for the BPMV-0 and BPMV-GFP constructs respectively.

**Table 1:** ***Bean pod mottle virus*** **(BPMV)-derived constructs used in this study**

**Name of the viral vector**	**RNA1-derived plasmid (pRNA1)**	**RNA2-derived plasmid (pRNA2)**
BPMV-0	pBPMV-IA-R1M^a^	pBPMV-IA-V1^a^
BPMV-GFP	pBPMV-IA-R1M^a^	pBPMV-GFP2^a^
BPMV-GmPDS	pBPMV-IA-R1M^a^	pBPMV-PDS-3R^a^
BPMV-PvPDS-52 bp	pBPMV-IA-R1M^a^	pBPMV-PvPDS-52 bp
BPMV-PvPDS-132 bp	pBPMV-IA-R1M^a^	pBPMV-PvPDS-132 bp
BPMV-PvPDS-262 bp	pBPMV-IA-R1M^a^	pBPMV-PvPDS-262 bp
BPMV-PvPDS-391 bp	pBPMV-IA-R1M^a^	pBPMV-PvPDS-391 bp

^a^DNA plasmids obtained from C. Zhang (Iowa State University, USA) [10]. *Abbreviations:* *BPMV* *Bean pod mottle virus*, *GFP* Green fluorescent protein, *Gm* *Glycine max*, *PDS* phytoene desaturase, *Pv* *Phaseolus vulgaris*.

Optimal plasmid quantity was determined using the BPMV-0 vector. To assess infection success, we used the pBPMV-IA-R1M plasmid carrying a mutated RNA1 as it is known to induce obvious moderate symptoms upon inoculation with pBPMV-IA-V1 compared with the symptomless WT RNA1, allowing the identification of infected plants by a simple visual inspection at 28 dpi [10]. We compared the number of plants displaying viral symptoms at 28 dpi after rub-inoculation with different quantities of RNA1- and RNA2-derived plasmids. In two independent experiments, 92%-100% of plants exhibited viral symptoms when inoculated with a plasmid DNA mix containing 5 μg of each plasmid, compared with 17% and 33% of those inoculated with 1.5 μg or 3 μg of each plasmid respectively (Table 2). Consequently, all further experiments were carried out using 5 μg of RNA1- and 5 μg of RNA2-derived plasmids. We also investigated whether rubbing intensity and the number of primary leaves inoculated (one or two) affected the infection rate in *P. vulgaris* cv. Black Valentine. We found that high-intensity rubbing (in 6 inoculated plants) resulted in injured areas on the upper leaf surface and no visible signs of infection at 28 dpi. Plants inoculated using low- or medium-intensity rubbing resulted in better infection rates (data not shown). There was no significant difference in the number of infected plants after rub-inoculation of either one or two primary leaves (data not shown). Therefore, the optimal conditions for direct rub-inoculation in *P. vulgaris* cv. Black Valentine were defined as: 5 μg of each RNA1- and RNA2-derived plasmid in a 20 μL mock buffer solution, and medium-intensity rubbing on one of the two primary leaves per plant.

**Table 2:** **Infection rates obtained after rub-inoculation with various quantities of RNA1- and RNA2-derived plasmids**

**Viral vector**	**Quantity of RNA1- and RNA2-derived plasmids (μg)**	**Infection rate** ^**a**^
BPMV-0	(1.5 + 1.5)	17% (1/6)
	(3 + 3)	33% (2/6)
	(5 + 5)	92-100% (11/12, 12/12)
BPMV-GFP	(5 + 5)	55% (5/9)
BPMV-GmPDS	(5 + 5)	33% (4/12)
BPMV-PvPDS-52 bp	(5 + 5)	50% (6/12)
BPMV-PvPDS-132 bp	(5 + 5)	67% (8/12)
BPMV-PvPDS-262 bp	(5 + 5)	67% (8/12)
BPMV-PvPDS-391 bp	(5 + 5)	58-91% (7/12,10/11)

^a^number of infected plants at 28 days post-inoculation/total of plants inoculated.

**Figure 1:**
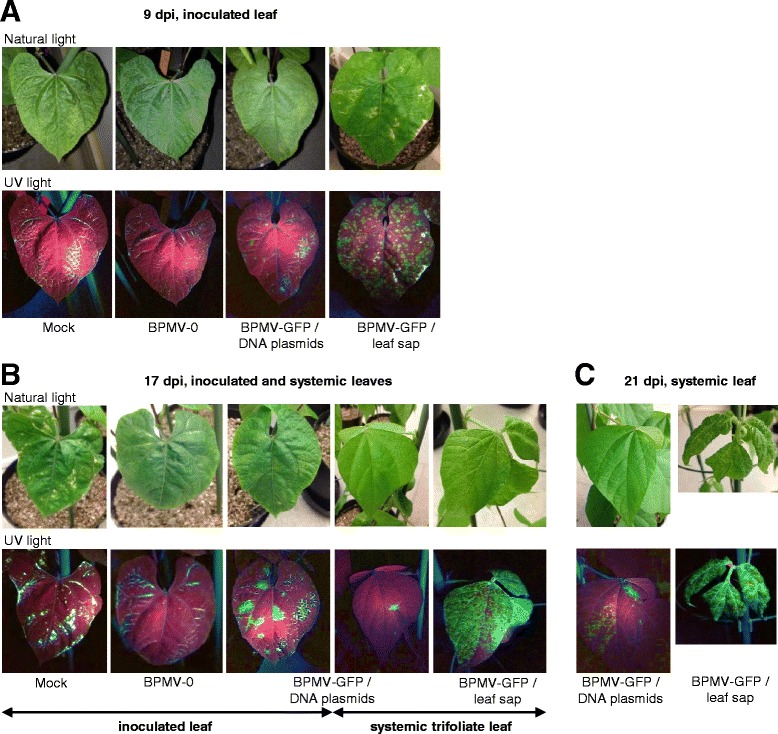
***Bean pod mottle virus*** **(BPMV)-induced expression of the** ***green fluorescent protein*** **(** ***GFP*** **) gene in leaves of** ***P. vulgaris*** **cv. Black Valentine after rub-inoculation with either infectious-DNA plasmids or leaf sap. (A-C)** GFP fluorescence in the primary-inoculated leaf **(A)**, in the primary and in third trifoliate leaf **(B)**, and in the third trifoliate (BPMV-GFP/DNA plasmids) or fourth trifoliate leaf (BPMV-GFP/leaf sap) **(C)** at nine, 17 and 21 days post-inoculation (dpi), respectively. Leaves of plants inoculated with mock buffer, BPMV empty vector (BPMV-0) or *GFP*-expressing vector (BPMV-GFP) were visualized under natural light (top panel) and UV light (bottom panel) and photographed. Similar results were obtained from three independent experiments.

To determine whether the insertion of a foreign gene fragment in BPMV RNA2 could affect the infection efficiency of BPMV during primary inoculation of *P. vulgaris* cv. Black Valentine, we used the BPMV-GFP vector which has a 720 bp fragment corresponding to the full-length GFP ORF inserted in RNA2 [10]. Seedlings were rub-inoculated with a DNA plasmid mix corresponding to BPMV-GFP (Table 1) following the conditions defined above. The first occurrence of GFP fluorescence was visible in leaves inoculated with BPMV-GFP at ~9 dpi (Figure 1A). Fluorescence pattern, in the form of round green spots, corresponded to the primary infection sites. As expected, no fluorescence was detected in the negative controls (plants inoculated with mock buffer or BPMV-0) (Figure 1A). At 17 dpi, the area displaying fluorescence had increased in the inoculated primary leaf and had extended to the third trifoliate leaves, indicating that the viral vector has moved to the upper systemic leaves (Figure 1B). Systemic infection of the third trifoliate leaves increased at 21 dpi (Figure 1C). At 28 dpi, 55% of the plants inoculated with BPMV-GFP were effectively infected (Table 2).

### *Temporal and spatial BPMV infection patterns in vegetative and reproductive tissues of* P. vulgaris *cv. Black Valentine*

Infection patterns were investigated in both vegetative and reproductive tissues of *P. vulgaris* cv. Black Valentine using GFP expression as a marker of infection. Seedlings were rub-inoculated with leaf sap derived from plants infected with BPMV-GFP. In the inoculated leaf, GFP fluorescence appeared 4–5 dpi, which was earlier than in DNA plasmid-infected plants. At 9 dpi, these leaves displayed extensive fluorescence, appearing as regularly distributed green round spots corresponding to the primary infection sites (Figure 1A). In the upper systemic leaves, the third and fourth trifoliates showed extensive green fluorescence at 17 and 21 dpi respectively, indicating that systemic infection occurred more rapidly than in DNA plasmid-infected plants (Figures 1B and C). High levels of fluorescence were also detected in stems (data not shown) and lateral roots (Additional file 1: Figure S1). At 4 wpi, 100% of the BPMV-GFP inoculated plants were infected, and similar results were obtained for BPMV-0 infected plants, demonstrating the high efficiency of viral infection using leaf sap. We also demonstrated that the BPMV-GFP vector was stable after four serial inoculations of *P. vulgaris* cv. Black Valentine (Additional file 1: Figure S2).

**Figure 2:**
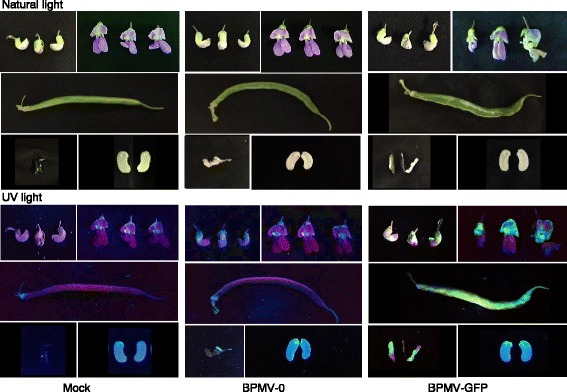
***Bean pod mottle virus*** **(BPMV)-induced expression of the** ***green fluorescent protein*** **(** ***GFP*** **) gene in reproductive tissues after rub-inoculation of one primary leaf with leaf sap.** Floral buds, flowers, pods and seeds of *P. vulgaris* cv. Black Valentine plants infected with mock buffer, BPMV empty vector (BPMV-0) and *GFP*-expressing vector (BPMV-GFP) were photographed at 30 days post-inoculation (pi), 30 days pi, 8 weeks pi and 10 weeks pi, respectively, under natural light (top panel) and UV light (bottom panel).

GFP fluorescence was detected in reproductive tissues of *P. vulgaris* cv. Black Valentine (Figure 2). Fluorescence was observed at 30 dpi in floral buds of BPMV-GFP infected plants. In petals, we observed stronger fluorescence in the standard (dorsal petal) compared with the lateral and ventral petals (Figure 2). At 8 wpi, pods of BPMV-0 and BPMV-GFP infected plants exhibited strong viral symptoms characterized by a curved shape and a bloated and mottled pod surface (Figure 2). When observed under UV light, infected pods from BPMV-GFP infected plants displayed extensive and homogenous GFP fluorescence (Figure 2). Notably, at 10 wpi, no GFP fluorescence was detected in the embryos of seeds harvested from BPMV-GFP-infected plants, while strong fluorescence was observed in the corresponding seed coats (Figure 2).

### *BPMV infection efficiency in other* P. vulgaris *cultivars*

As VIGS is an effective genomics tool only in genotypes where the viral vector can spread systemically, we tested different *P. vulgaris* cultivars (JaloEEP558, BAT93, G19833, DOR364, TU and La Victoire) for their susceptibility to BPMV. Of significant interest are JaloEEP558 and BAT93, the two parental lines of a RILs population used to set up the integrated linkage map of *P. vulgaris* [29], and BAT93 and G19833 whose complete genomes have been sequenced [2]. Black Valentine was included as a control of susceptibility to BPMV.

**Figure 3:**
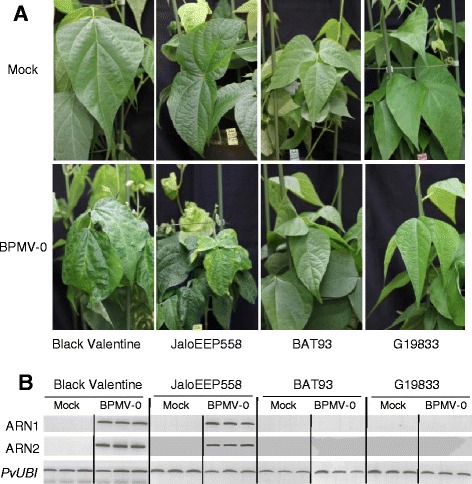
**Screening of** ***P. vulgaris*** **cultivars for susceptibility to** ***Bean pod mottle virus*** **(BPMV). (A)** Mock inoculated plants (top panel) and BPMV-0 inoculated plants (bottom panel) were photographed under natural light at 28 days post-inoculation (dpi). For BPMV-0, mechanical inoculation was made by rubbing of infected leaf sap. **(B)** Semi-quantitative RT-PCR of BPMV RNA1 and RNA2 in mock- and BPMV-0 treated plants. Ubiquitin (*PvUBI*) was used as an internal control. Total RNA was extracted at 21 dpi from the third trifoliate leaf of three plants for Black Valentine, BAT93 and G19833 and from the second trifoliate leaf for JaloEEP558.

The three genotypes of significant interest were first inoculated with leaf sap containing the BPMV-0 vector (Table 1) [10]. As in Black Valentine, upper systemic leaves of infected JaloEEP558 plants displayed strong viral symptoms at 28 dpi (Figure 3A). By contrast, systemic leaves of infected BAT93 and G19833 plants were symptomless at 28 dpi and looked like systemic leaves of mock-inoculated plants (Figure 3A). Semi-quantitative RT-PCR on systemic leaves of mock- and BPMV-0-inoculated plants with primers specific to BPMV RNA1 and RNA2 confirmed that viral RNAs were present only in systemic leaves of JaloEEP558 plants inoculated with BPMV-0 (Figure 3B). No viral RNA was amplified in the systemic leaves of BAT93 and G19833 (Figure 3B).

**Figure 4:**
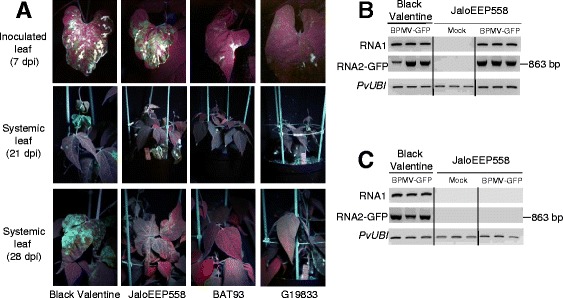
***Bean pod mottle virus*** **(BPMV)-induced expression of the** ***green fluorescent protein*** **(** ***GFP*** **) gene in leaves of** ***P. vulgaris*** **genotypes of interest. (A)** BPMV-GFP inoculated plants were photographed under UV light, at 7 days post-inoculation (dpi) for inoculated leaves, and at 21 dpi and 28 dpi for systemic leaves. **(B-C)** Semi-quantitative RT-PCR of BPMV RNA1 and RNA2 in inoculated leaves **(B)** and systemic leaves **(C)** of control plants (mock treatment) and plants inoculated with BPMV-GFP. Ubiquitin (*PvUBI*) was used as an internal control. Total RNA was extracted at 9 dpi from the inoculated leaves and at 30 dpi from the fourth trifoliate and third trifoliate leaves of three different plants of Black Valentine and JaloEEP558, respectively.

All genotypes were then tested using the BPMV-GFP vector. We detected fluorescence in inoculated leaves at 7 dpi only in the JaloEEP558 cultivar, and to a lesser extent in the G19833 and La Victoire cultivars (Figure 4A and Additional file 1: Figure S3). When compared to inoculated leaves of Black Valentine, the intensity of GFP fluorescence was greater in inoculated leaves of JaloEEP558 (Figure 4A). Surprisingly, systemic leaves of JaloEEP558 did not display GFP fluorescence at 21 or 28 dpi. This failure of long-distance movement is not intrinsic to the BMPV-GFP construct, as it has been found to be capable of long-distance movement in Black Valentine (Figure 4A). GFP expression was confirmed by semi-quantitative RT-PCR with primers specific to both BPMV RNAs. For RNA2, specific primers were designed to span the cloning site of the *GFP* ORF and produced a PCR product of 863 bp in inoculated leaves of both Black Valentine and JaloEEP558 plants treated with BPMV-GFP (Figure 4B). By contrast, no corresponding PCR product was amplified in systemic leaves of JaloEEP558 inoculated with BPMV-GFP (Figure 4C). Furthermore, no RNA2 band of lower size was visible on the electrophoresis gel after amplification with RNA2-GFP primers on samples of JaloEEP558 systemic leaves (data not shown), excluding an eventual recombination within RNA2 of BPMV-GFP resulting in an entire or partial loss of the *GFP* ORF.

### *Virus-induced gene silencing of PvPDS in* P. vulgaris *cv. Black Valentine using a heterologous gene fragment*

**Figure 5:**
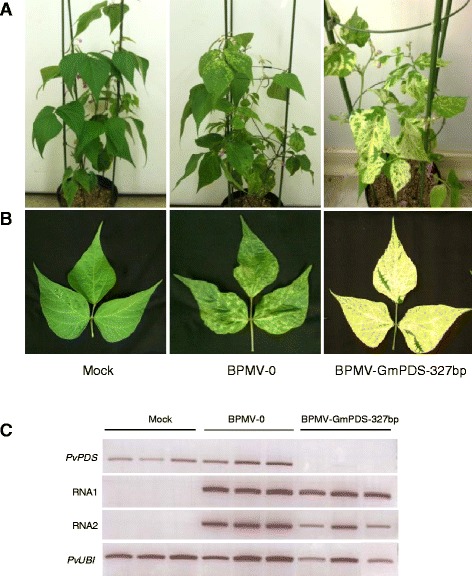
**Silencing of** ***PDS*** **in** ***P. vulgaris*** **cv. Black Valentine using the VIGS vector BPMV-GmPDS-327 bp. (A-B)** Plants inoculated with mock buffer (left panel), BPMV-0 (middle panel), and BPMV-GmPDS-327 bp (right panel) were photographed under natural light at 28 days post-inoculation (dpi). **(B)** Trifoliate systemic leaves. **(C)** Semi-quantitative RT-PCR of *PvPDS*, BPMV RNA1 and RNA2 in systemic leaves of plants inoculated with mock, BPMV-0, and BPMV-GmPDS-327 bp. Ubiquitin (*PvUBI*) was used as an internal control. Total RNA was extracted at 21 dpi from the third trifoliate leaf of three different plants of Black Valentine.

The efficiency of endogenous gene silencing using the BPMV VIGS vector delivered through direct DNA rubbing in *P. vulgaris* cv. Black Valentine was investigated by targeting the *PvPDS* gene. *PDS* is routinely used as a marker gene for VIGS in plants as silencing this gene causes chlorophyll degradation resulting in a typical photobleached phenotype in emerging leaves. Initial tests were carried out with the BPMV-GmPDS-327 bp construct containing a 327 bp fragment of the *PDS* gene from *Glycine max* (*GmPDS*) (Table 1) as it was immediately available (supplied by C. Zhang) [10]. Alignment of the 327 bp *GmPDS* fragment with *PvPDS* sequences from G19833 and BAT93 revealed a high level of sequence conservation with 5 DNA stretches of 23 nt or more (the minimal length for VIGS induction, [30]) having 100% identity between the two *PvPDS* sequences and the *GmPDS* sequence (Additional file 1: Figure S4). The BPMV-GmPDS-327 bp construct was delivered into *P. vulgaris* cv. Black Valentine seedlings by direct rub-inoculation. Infected leaves were used for secondary inoculations of healthy plants. The infected plants displayed photobleached leaves at 28 dpi, unlike plants infected with the empty BPMV-0 vector or mock buffer (Figures 5A and B).

In order to confirm that the photobleached phenotype described above correlated with reduced endogenous levels of *PvPDS*, semi-quantitative RT-PCR was carried out on systemic leaves from each of the three treatment groups (Figure 5C). To test whether the phenotype observed in treated plants could be due to the presence of the viral vectors, the presence of BPMV RNA1 and RNA2 transcripts was also determined by RT-PCR (Figure 5C, middle 2 gels). As expected, samples from the mock-treated plants did not show viral RNA1 and RNA2 unlike BPMV-0 and BPMV-GmPDS-327 bp inoculated plants (Figure 5C). BPMV-0 inoculated plants showed expression levels of *PvPDS* similar to that of mock-treated plants, suggesting that the viral treatment does not interfere with *PvPDS* expression (Figure 5C). In samples from the BPMV-GmPDS-327 bp treated plants, there was a strong down-regulation of *PvPDS* (relative to ubiquitin), as indicated by the lack of visible bands on the gel (Figure 5C).

### *Minimal fragment size for efficient VIGS of* PvPDS *in* P. vulgaris *cv. Black Valentine*

To determine the minimal size required to induce efficient silencing by the BPMV-derived vector, fragments ranging in size from 52 to 391 bp (Table 1) of the PvPDS gene from JaloEEP558 were cloned into the *Bam*HI restriction site of the pBPMV-IA-V1 plasmid. The different fragment sizes ranging from 52 to 391 bp were chosen in the same 3’-end coding region of the *PvPDS* gene. The fragment of 52 bp corresponds to the longest region presenting 100% nucleic identity between the 327-bp fragment of the *Glycine max PDS* ortholog (*GmPD*S) and the corresponding regions of *PvPDS* from *P. vulgaris* cv. G19833 (*PvaPDS*) and BAT93 (*PvmPDS*) (Additional file 1: Figure S4). The fragment of 391 bp corresponds approximately to the insert size chosen by Zhang *et al.* [10]. The two fragments of 132 and 262 bp present intermediate sizes between 52 and 391 bp.

**Figure 6:**
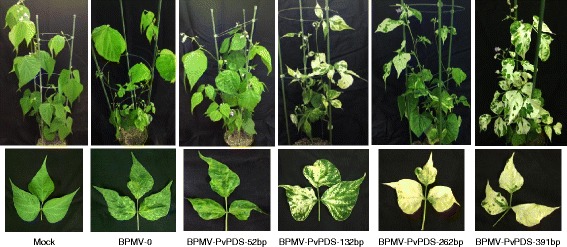
**Virus-induced gene silencing of** ***PvPDS*** **in** ***P. vulgaris*** **cv. Black Valentine using the BPMV-derived vector containing fragments of various sizes.** Plants inoculated with mock buffer (first left panel), BPMV-0 (second panel), and BPMV-PvPDS-52 bp to BPMV-PvPDS-391 bp were photographed under natural light at 28 days post-inoculation (dpi). Representative trifoliate leaves of the corresponding plants are shown in the lower panel.

Homologous 391 bp-region of PvPDS from JaloEEP558 and Black Valentine were 100% identical (data not shown). Plasmids containing *PvPDS* gene fragments of different lengths were used for primary inoculation of *P. vulgaris* cv. Black Valentine seedlings, which were then used for secondary inoculation of wild type plants. At 4 wpi, plants inoculated with the BPMV-PvPDS-262 bp and BPMV-PvPDS-391 bp constructs displayed a clear photobleached phenotype with completely white newly emerging leaves (Figure 6). No photobleaching was observed in plants inoculated with BPMV-PvPDS-52 bp (Figure 6). Plants inoculated with BPMV-PvPDS-132 bp displayed an intermediate phenotype characterized by green leaves with white sectors (Figure 6). This result demonstrates that a fragment of 132 bp, bearing 100% homology with the targeted sequence, is sufficient to trigger efficient silencing of an endogenous gene by the BPMV-derived vector in common *bean* cv. Black Valentine. Nevertheless, as VIGS efficiency throughout the plant was higher with the BPMV-PvPDS-391 bp vector, further experiments were conducted using this vector.

The duration of VIGS was estimated in *P. vulgaris* cv. Black Valentine plants inoculated with the BPMV-PvPDS-391 bp vector. Plants grown under normal light conditions showed recovery of silenced leaves more than 2 months pi, as characterized by an overall decline of white leaves over time. However, silenced plants placed under high intensity illumination (sodium lamp) displayed a photobleached phenotype for more than 3 months pi (data not shown).

### *Virus-induced gene silencing of* PvPDS *in* P. vulgaris *cv. JaloEEP558*

To evaluate the efficiency of *PvPDS* VIGS in JaloEEP558, rub-inoculation was carried out with the BPMV-PvPDS-391 bp vector derived from leaf sap extracted from infected leaves of primary inoculated Black Valentine plants.

**Figure 7:**
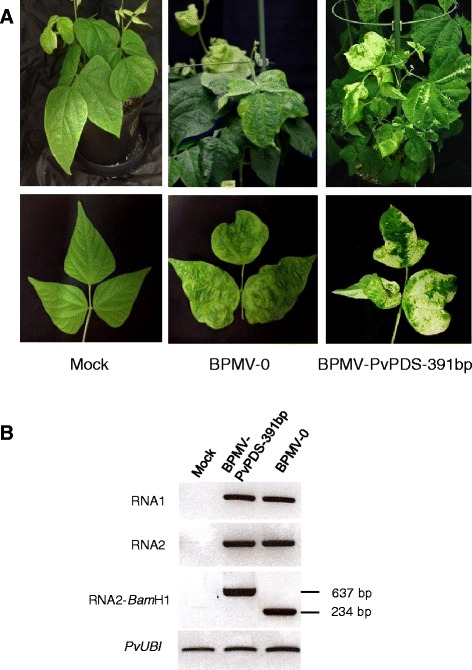
**Virus-induced gene silencing of** ***PvPDS*** **in** ***P. vulgaris*** **cv. JaloEEP558 using the BPMV-PvPDS-391 bp vector. (A)** Plants inoculated with mock buffer (first left panel), BPMV-0 (middle panel), and BPMV-PvPDS-391 bp were photographed under natural light at 7 weeks post-inoculation (wpi). Representative trifoliate leaves of the corresponding plants are represented in the lower panel. **(B)** Semi-quantitative RT-PCR of BPMV RNA1 (upper panel) and RNA2 (two middle panels) in systemic leaves of plants inoculated with mock buffer, BPMV-PvPDS-391 bp and BPMV-0. Ubiquitin (*PvUBI*) was used as an internal control. Total RNA was extracted at 7 wpi from a pool of three fourth trifoliate leaf of three different plants of JaloEEP558.

The onset of silencing, with the appearance of photobleaching, was delayed in JaloEEP558 (at ~7 wpi) compared to Black Valentine (at ~4 wpi). Moreover, complete whitening of trifoliate leaves was less frequent in JaloEEP558 than in Black Valentine controls, and in most cases, intermediate phenotypes were observed with leaflets having white sectors or whitening limited to the vasculature (Figure 7A).

Although photobleaching was limited, systemic leaves of JaloEEP558 exhibited typical viral symptoms (Figure 7A). Thus, to be sure that systemic leaves with viral symptoms still contained the BPMV RNA2 carrying the *PvPDS* 391-bp insert, we performed semi-quantitative RT-PCR analyses. RT-PCR with RNA2-specific primers spanning the *Bam*HI cloning site produced a product size of 234 bp in leaves of plants inoculated with BPMV-0, corresponding to the distance between primers in the absence of insert (Figure 7B). By contrast, a larger PCR product of 637 bp was amplified in samples of BPMV-PvPDS-391 bp-inoculated plants ,thereby confirming the presence of the 391-bp *PvPDS* insert within BPMV RNA2 in the systemic leaves of JaloEEP558 (Figure 7B).

We also tested the BPMV-PvPDS-262 bp and BPMV-PvPDS-132 bp vectors in JaloEEP558. No enhanced silencing phenotype was observed compared to plants inoculated with BPMV-PvPDS-391 bp (data not shown), although these vectors also spread systemically in JaloEEP558 without losing their *PvPDS* insert (data of RT-PCR analyses not shown).

### Phenotyping of the resistance to BPMV in common bean RILs and genetic mapping of the R-BPMV gene

**Figure 8:**
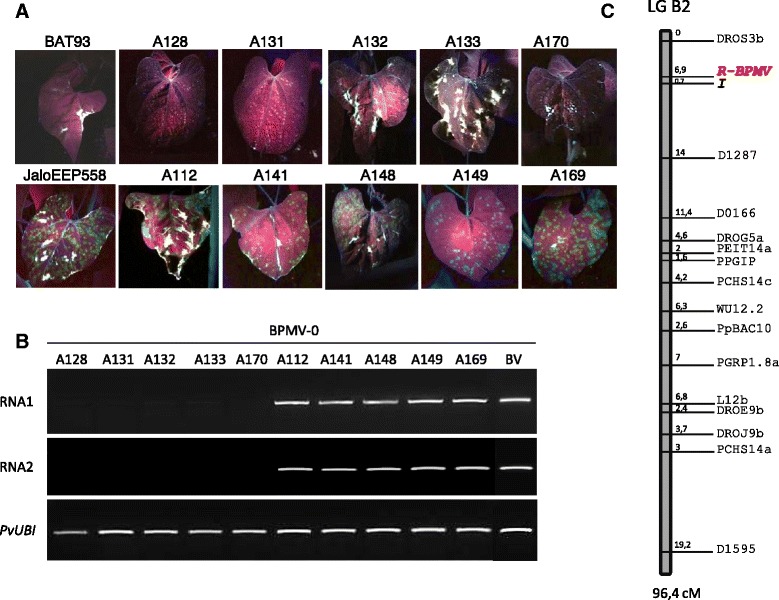
**Phenotyping the resistance to** ***Bean pod mottle virus*** **(BPMV) in** ***P. vulgaris*** **RILs and genetic mapping of the** ***R-BPMV*** **gene on the integrated linkage map. (A)** Phenotyping the resistance to BPMV was achieved in BPMV-GFP inoculated plants by visual inspection of inoculated leaves under UV light at 7 days post-inoculation (dpi). Representative inoculated leaves are shown for the two parental lines (BAT93 and JaloEEP558) and a sample of 10 RILs. **(B)** Semi-quantitative RT-PCR of BPMV RNA1 and RNA2 in systemic leaves of 10 RILs inoculated with the empty BPMV vector (BPMV-0). Ubiquitin (*PvUBI*) was used as an internal control. Total RNA was extracted at 28 dpi from a pool of three trifoliate leaves from three different plants of each RIL. **(C)** Location of *R-BPMV* on LG B2 based on the 77 BAT93 X JaloEEP558 RILs population [29,44,45]. Marker names are indicated on the right of the linkage group. Map distances are indicated between molecular markers and expressed in Kosambi centiMorgans.

Our finding that the parental genotypes of a 77 RILs population used to set up the integrated linkage map of common bean [29] differed markedly in their susceptibility to BPMV (JaloEEP558 was susceptible to BPMV-0 and BPMV-GFP, whereas BAT93 was resistant with no replication of BPMV-0 and BPMV-GFP in either inoculated or systemic leaves) allowed to us to investigate the genetic control of BPMV resistance. The 77 RILs inoculated with BPMV-GFP were phenotyped at 7 dpi. Presence of fluorescent local lesions on the inoculated leaf was scored as “susceptible” (JaloEEP558 type) and absence of GFP fluorescence was scored as “resistant” (BAT93 type) (Figure 8A). The observed segregation ratio fitted a 1:1 ratio of susceptible to resistant plants (χ2 = 0.373, P = 0.54) suggesting that a single gene (*R-BPMV*) is segregating.

The BPMV-GFP construct is an ideal tool to phenotype the 77 RILs since it allowed a visual, rapid and non-destructive scoring of resistance to BPMV. To test whether the presence of the GFP ORF in the BPMV RNA2 could interfere with resistance/susceptibility to BPMV, we chose a set of 5 resistant and 5 susceptible RILs (A128, A131, A132, A133, A170 and A112, A141, A148, A149, A169, respectively) (Figure 8A) and inoculated them with the empty vector construct (BPMV-0) or mock buffer. At 28 dpi, trifoliate leaves of all 10 RILs were visually inspected for the presence of viral symptoms relative to BPMV systemic infection. As expected, all 5 resistant RILs were symptomless, as were the mock-treated plants (data not shown). Among the 5 susceptible RILs, all displayed viral symptoms, except A149 which looked like mock-inoculated plants (data not shown). Three trifoliate leaves from three different plants of each RIL were harvested at 28 dpi and pooled. RT-PCR analyses were performed on these leaf pools using BPMV RNA1- and RNA2-specific primers (Figure 8B). No viral RNAs were detected in any of the 5 resistant RILs whereas viral RNAs were amplified from all 5 susceptible RILs (Figure 8B). These results confirmed that common bean resistance to BPMV can be scored using the BPMV-GFP construct, instead of wild-type BPMV.

Using the total 77 RILs, the *R-BPMV* gene was mapped at one end of LG B2, between marker DROS3b and the *I* locus, at 6.9 cM and 0.7 cM respectively (Figure 8C). The *I* locus has been previously shown to control the development of four different phenotypes in response to inoculation with several potyviruses [31,32], and one comovirus (*Bean severe mosaic virus*, BSMV) [33,34].

## Discussion

In the post-genomic era, increasing efforts are being made in plant functional genomics. VIGS technology is a simple and powerful tool that has been widely used to analyze gene function in many plant families such as *Solanaceae*, *Brassicaceae*, *Poaceae*, *Ranunculaceae*, and *Asteraceae* (reviewed in [11]) and especially *Fabaceae* [7] where many species are difficult to transform genetically by other means. Recent improvements in VIGS methodology have been reported such as the development of new VIGS vectors, a widening of the viral host range, and the improvement of vector delivery methods [7,11]. The development of direct rub-inoculation of column–purified plasmids has simplified the inoculation procedure, making it rapid and cost-effective for high-throughput functional analyses [8-10]. Rub-inoculation was found to be similarly effective to biolistic delivery in soybean, with infection rates ranging from 50-58% (average 54%) for direct DNA rubbing and 50-80% (average 65%) for biolistic inoculation [10,27]. Here we show for the first time that direct DNA rubbing, using a “one-step” BPMV vector originally developed in soybean, is a convenient delivery procedure in common bean. Infection rates in common bean cv. Black Valentine, ranging from 33-91% (average 60%) for primary inoculations and 100% for secondary inoculations using leaf sap, were comparable to those obtained in soybean by either DNA rubbing or biolistic delivery. Previous work by Diaz-Camino *et al.* [28] demonstrated efficient VIGS in cv. Black Valentine using the first generation BPMV vector [26] with a 40-80% infection rate (average 60%) for primary inoculations and 100% for secondary inoculations. However, this vector has limiting features, including the requirement for vector delivery to be carried out by mechanical inoculation of *in vitro*-transcribed RNA, which is a rather labor intensive and an expensive procedure. The infection rates reported here are comparable to those reported in Diaz-Camino *et al.* [28], with the advantage of using a simplified, cost-effective method that is still highly consistent. Advantages of the “one-step” BPMV vector in common bean include: (1) no *in vitro* transcription of plasmid DNA required, (2) vector delivery into plants by conventional rub-inoculation, without any requirement for biolistic or agro-inoculation procedures, and (3) silencing induced in 60% of the inoculated plants with low quantities (five μg) of each infectious plasmid carrying BPMV RNA1 and RNA2. These features are critical for the development of high-throughput infection procedures.

Plant virus-based vectors are valuable tools for heterologous protein expression in plants. These vectors are fast-acting, cost-efficient, high yield, and significantly they can be used in a variety of genetic backgrounds provided that a given genotype is susceptible to the viral vector. To date, the BPMV vector is the only available virus-based system that is effective for stable expression of foreign protein in common bean. We found that a recombinant BPMV-GFP vector gives an intense GFP expression in a large range of vegetative and reproductive organs in common bean cv. Black Valentine (Figures 1, 2 and Additional file 1: Figure S1). In the “one-step” BPMV vector used in this study, the foreign ORF (e.g. GFP) is inserted before a foot and mouth disease virus (FMDV) 2A proteinase peptide engineered between the movement protein and the large coat protein (L-CP) cistrons of RNA2 [10]. Because the cleavage at the 2A site occurs between the two last amino acids, the foreign protein contains ~20 additional amino acids and the L-CP has an additional proline at its N-terminus. These characteristics have led Diaz-Camino *et al.* [28] to suggest that such a recombinant vector could be difficult to maintain through serial passages in plants. However, our results showed that GFP expression was stable after four serial passages in cv. Black Valentine (Additional file 1: Figure S2), suggesting that the “one-step” vector is as efficient as the first-generation vector which was also stable after four serial passages in both soybean cv. Essex and common bean cv. Black Valentine [26,28]. Further investigations are needed to determine the maximal insert size compatible with virus infectivity and systemic movement for a given genotype. Previous studies have estimated the BPMV RNA2 vector capacity for foreign gene insertion to be between 1.4 and 1.8 kb [10,26]. Although the average length of a plant gene is ~3 kb, it could be possible to express short proteins such as bacterial or fungal effectors involved in plant immune responses.

Endogenous gene silencing is an important tool for functional genetics. As common bean is not amenable to stable genetic transformation, VIGS provides a rapid and powerful tool to dissect gene function in this economically important species. In this study, we found that the VIGS method using the “one-step” BPMV vector induced efficient silencing of the endogene *PvPDS* in common bean cv. Black Valentine (Figures 5 and 6). Down-regulation of *PvPDS* transcripts in infected Black Valentine plants was nearly total (Figure 5C). Moreover, the photobleached phenotype linked to *PvPDS* down-regulation was stable under our experimental conditions, lasting for 2 months in plants grown under normal light conditions and for 3.5 months in plants grown under high intensity illumination. In barley, reversion of *PDS* silencing has been shown to be caused by major deletions in the *PDS* insert carried by a *Barley stripe mosaic virus* (BSMV)-derived vector [35]. In BSMV-infected barley plants, a 400 bp-*PDS* insert was partially lost at 7 dpi and completely lost at 4 wpi. By contrast, there was no evidence of deletions at 7 wpi in JaloEEP558 plants infected with BPMV-PvPDS-391 bp (Figure 7B), confirming the stability of the “one-step” BPMV vector.

Our results showed that BPMV spreads systemically throughout the whole plant in Black Valentine (Figures 1, 2 and Additional file 1: Figure S1). Following BMVP-GFP infection, fluorescence was detected in primary leaves, trifoliate leaves, stems, roots, floral buds, flowers, pods, and seed coats (Figures 1, 2 and Additional file 1: Figure S1). As VIGS is effective only in the tissues infected by the viral vector, it should therefore be possible to induce gene silencing in all these organs.

One limitation of VIGS is that it is possible only in genotypes where the viral vector can spread systemically. For a given species, different genotypes might react differently to viral infection, from total resistance due to specific resistance genes to various levels of susceptibility. This has been well-documented for example in the *Pea early browning virus*-derived vector in *Medicago truncatula* and *Lathyrus odorata* [36]. So, it is essential to test the susceptibility of each genotype of interest. Prior to this study, Black valentine was the only common bean genotype known to be susceptible to BPMV [10,28]. We analyzed six other common bean genotypes and found that five (BAT93, G19833, DOR364, TU, and La Victoire) were resistant to BPMV (Figures 3, 4 and Additional file 1: Figure S2), and only one (JaloEEP558) was susceptible. This implies that VIGS using the “one-step” BPMV vector will not be possible in the two sequenced genotypes of common bean (BAT93 and G19833). In JaloEEP558, VIGS was shown to be delayed compared to Black Valentine and displayed a patchier phenotype. Thus, implementation of VIGS in JaloEEP558 will require further investigation to improve VIGS efficiency. The extent to which the “one-step” BPMV vector can be used in other legume species is unknown, and it would be interesting to test the susceptibility to BPMV in species such as *Cajanus cajan* or *Cicer arietinum*, whose genomes have recently been sequenced [37,38], *Pisum sativum*, another economically important legume species for human consumption and animal feeding in Europe, as well as the model legume *Medicago truncatula* [39].

To improve VIGS analysis in common bean, some technical improvements might be considered. First, as BPMV viral symptoms could interfere with the silencing phenotype of a given target gene, it should be possible to use the wild type RNA1 from isolate IA-Di1 rather than the mutated variant RNA1M (which induces moderate visible viral symptoms) used in this study. Second, VIGS in target tissues is usually patchy due to the irregular distribution of the vector in the whole plant. In order to identify infected areas undergoing silencing, *PDS* could be used as a traceable marker that would be silenced along with the target gene. A recombinant BPMV vector containing a *PDS* insert in addition to the target gene insert could be engineered based on the pBPMV-IA-V2 construct designed by Zhang *et al.* [10]. In this construct, the *Bam*H1 restriction site is replaced by a multicloning site so that simultaneous silencing of two endogenes could be achieved. We found that *PDS* silencing can be achieved with an insert of only 132 bp resulting in a patchy photobleached phenotype (Figure 6). Combination of the 132 bp-PDS insert with a second insert corresponding to the target gene could therefore be a good compromise for conducting marker-assisted VIGS experiments. However, additional experiments need to be carried out to evaluate any possible impact of *PDS* silencing on unintended target gene expression. Such interference has been described in *Gerbera hybrid*, where photobleaching induced by a *Tobacco rattle virus* (TRV)-PDS construct resulted in silencing of the polyketide synthase gene *G2PS1* involved in the anthocyanin synthesis pathway, which has no apparent link to the carotenoid biosynthesis pathway [40].

In this study, we reported the genetic mapping of an R-gene (*R-BPMV*) present in the BPMV-resistant BAT93 and absent in the BPMV-susceptible JaloEEP558, which confers an extreme resistance to BPMV isolate IA-DI1. Using the BMPV-GFP construct as a marker of susceptibility, we found that phenotypic segregation of the 77 RILS derived from a cross between BAT93 and JaloEEP558 [29] fitted a 1:1 ratio, suggesting that a single gene may be implicated in viral resistance. As far as we know, this is the first report in plants of genetic mapping of a virus resistance gene using a recombinant viral vector expressing an easily traceable marker such as the GFP protein. Main advantages of using the BPMV-GFP construct for phenotyping RILs include rapid (on 19 day-old seedlings) and highly reliable results that preclude the need for molecular analyses such as RT-PCR that would be required if symptoms were ambiguous.

Genetic mapping of the *R-BPMV* gene is significant for two reasons. First, it will allow the functional validation of other resistance genes present in BAT93. Indeed, BAT93 possesses many resistance genes against damaging pathogens of common bean such as *Colletotrichum lindemuthianum*, a hemibiotrophic fungus. Functional validation of these genes, for example *Co-9* located on linkage group 4 [41], cannot be monitored by BPMV-mediated VIGS studies, since BAT93 is resistant to BPMV. However, it would be possible to identify a RIL individual, derived from the cross between BAT93 and JaloEEP558, which carries both the BMPV susceptibility of JaloEEP558 and the *Co-9* gene from BAT93. In such a RIL genotype, VIGS is feasible and the resistance gene of interest is present and can therefore be silenced. The second area of interest is related to agricultural applications. BPMV has been reported to be a major threat to soybean production in the USA [24]. To our knowledge, all commercially available cultivars of soybean are susceptible to BPMV infection. We suggest that *R-BPMV* may be a useful resource for the development of BPMV resistant soybean, as the *R-BPMV* gene confers extreme resistance to BPMV isolate IA-Di1. We are currently fine-mapping the *R-BPMV* gene, located near the *I* locus previously implicated in virus resistance [31-34].

## Conclusions

In this paper, we report the successful implementation in common bean of a DNA-based BPMV vector originally developed in soybean by Zhang *et al.* [10]. We showed that the “one-step” BPMV vector has the potential to enable cost-effective and simplified screening of common bean plants via a simple “one-step” rub-inoculation of miniprep-quality plasmid DNA. This BPMV vector can be used for heterologous protein expression, target gene silencing by VIGS, and genetic mapping of a BPMV resistance gene in common bean. The “one-step” BPMV vector system enables rapid and simple functional studies in common bean, and is suitable for large-scale analyses. Our protocol could facilitate the characterization of the many genes in common bean, particularly genes involved in disease resistance. In the post-genomic era, these advances are timely for the common bean research community.

## Methods

### Plant materials and growth conditions

The following genotypes of *Phaseolus vulgaris* were used in this study: Black Valentine, JaloEEP558 (Andean landrace), BAT93 (Mesoamerican breeding line), G19833 (CIAT Germplasm accession), TU, DOR364, and La Victoire (French cultivar of Andean origin developed by the seed company “Tezier” (Valence-sur-Rhône, France)). Seeds were sown in vermiculite and grown in a growth chamber at 23°C under a 16 h light/8 h dark cycle and 75% relative humidity. Seedlings were cultivated in plastic pots filled with moist vermiculite during 10–12 days (the stage at which primary leaves are fully-expanded). Three seedlings were then transplanted in moist vermiculite in a 20 cm plastic pot and placed in a dark room 24 h prior to inoculation. For genetic mapping experiments, a population of 77 F11 RILs, derived from the cross between JaloEEP558 and BAT93 was used to map the *R-BPMV* resistance gene. Seedlings of the 77 RILs at the fully-expanded primary leaf stage were placed individually in a dark room 24 h prior to inoculation, and phenotyped 7 days post-inoculation (dpi).

### Viral vectors

Names of the BPMV constructs used in this study and the corresponding RNA1- and RNA2-derived plasmids are listed in Table 1. The pBPMV-IA-R1M, pBPMV-IA-V1, pBPMV-GFP2 and pBPMV-PDS-3R DNA plasmids were kindly supplied by C. Zhang from Iowa State University, USA, and were described in Zhang *et al.* [10]. All others RNA2-derived plasmids were constructed in our laboratory by insertion of *P. vulgaris* target gene fragments in the *Bam*H1 restriction site of pBPMV-IA-V1. For construction of BPMV-PvPDS-52 bp, we directly cloned a double-stranded synthetic oligonucleotide (Table 3) into the *Bam*H1 restriction site. For all other BPMV-derived vectors, target gene fragments were first amplified by PCR using specific primers (Table 3), *P. vulgaris* cDNAs as template, and a high fidelity polymerase (Advantage HF2, Clontech). PCR products were digested with *Bam*H1 and inserted into *Bam*H1-digested and dephosphorylated pBPMV-IA-V1 to generate the VIGS vectors listed in Table 1. The orientation of the cloned inserts was determined by PCR using a combination of vector-specific and fragment-specific primers (Table 3). Clones thought to contain inserted fragments in the antisense orientation were sequenced (GATC, Germany) to confirm their identity and were subsequently used for gene silencing.

**Table 3:** **PCR primers used for construction of the BPMV VIGS vectors and RT-PCR analyses**

**Name of primer**	**DNA sequence of primer (5′-3′)**	**Size of PCR product (bp)**
PvaPDS-F1	cgggatccAGCAGAAGTCCCCTTCTGAG	391
PvaPDS-R1	cgggatccCTTGTGCACACAGCTTCC	
PvaPDS-262fwd	cgggatccAGAATGGATTTCACGTAGT	262
PvaPDS-262rev	cgggatccAACAGCACCTTCCATTGAAGCT	
PvaPDS132-rev	cgggatccGGTGTTTTAACAACATGGTAC	132^a^
PvaPDS52-fwd	GATCGCTTTGCTTTGGTCTGCAGAAATTTCATCAGGAAAGAGTTTGGCAAGCTCAGT	-
PvaPDS52-rev	GATCACTGAGCTTGCCAAACTCTTTCCTGATGAAATTTCTGCAGACCAAAGCAAAGC	-
RNA1-fwd	CAAGCCCAAAGTGCTGAAGT	160
RNA1-rev	GCAAATCCAACTGCCATTCT	
RNA2-fwd	ATACCCCTAATGGCACAGGA	269
RNA2-rev	GGAAATGTAACCACCCGAAT	
RNA2-*Bam*H1-fwd	TGACAATCCCAAACAGTCTACAG	234
RNA2-*Bam*H1-rev	AGCATACTCAACGAGAGGGTCA	
RNA2-GFP-fwd	TGAGGTTCAGGCTCAGATGGA	863
RNA2-GFP-rev	GGCCTGGATTTGATTCTACA	
PvPDS-fwd	ACATCTTCTTTGGGGCTTACC	200
PvPDS-rev	CAGCATCTCGTTGTTCCTCA	
PvUBI-fwd	CAGCTGGAGGATGGAAGG	200
PvUBI-rev	TCCGAACTCTCCACCTCAAGA	

^a^in combination with PvaPDS-262fwd.

### Viral inoculation procedure

All plants were placed in the dark 24 h prior to inoculation as reported in *Arabidopsis thaliana* [8] and *Glycine max* [10]. For primary inoculation, a DNA-plasmid mix was prepared in 20 μL using 50 mM potassium phosphate buffer, pH 7.0 (=mock buffer). For secondary inoculations, an infected leaf of *P. vulgaris* cv. Black Valentine (fresh leaf or frozen leaf conserved at −80°C) was ground in a mortar with mock buffer to make leaf sap. Inoculations were performed by mechanical rubbing of one primary leaf (unless otherwise stated) following the procedure described by Pflieger *et al.* [8]. The inoculated plants were allowed to grow at 19°C under a 16 h light/8 h dark cycle in a growth chamber.

### *Optimization of conditions for rub-inoculation of infectious BPMV-derived plasmids in* P. vulgaris

*P. vulgaris* cv. Black Valentine plants at the fully-expanded primary leaf stage (10–12 days post-germination) were used in all experiments. The BPMV RNA1 plasmid was pBPMV-IA-R1M. The BPMV RNA2 plasmid was either pBPMV-IA-V1 or pBPMV-GFP2 (Table 1) [10]. A 20 μL plasmid DNA mix containing equal amounts (either 1.5, 3, or 5 μg) of pBPMV-IA-R1M and either pBPMV-IA-V1 or pBPMV-GFP2, was prepared using mock buffer. Three intensities of rubbing were evaluated: low-intensity (one passage of the gloved finger per leaf surface), medium-intensity (two passages) and high-intensity (three passages).

### GFP imaging

GFP expression in aerial organs and roots was examined using a UV lamp (High intensity 100-Watt long-wave UV lamp; UVP, USA) and photographed using a Canon PowerShot A2300 digital camera. Microscopic observations of roots were made at 21 dpi using an Axioskop microscope (Zeiss, Germany) and photographed using a RT_KE_ camera (SPOT, USA).

### Expression analysis by semi-quantitative RT-PCR

Total RNA and cDNA synthesis were performed as described in Richard *et al.* [42]. Primer sequences are listed in Table 3. Primers PvPDS-fwd and PvPDS-rev used to amplify the *PvPDS* gene were designed to anneal outside the region targeted for silencing, so that only the endogenous gene was probed. Primers PvUBI-fwd and PvUBI-rev were used to amplify *PvUBI* as an internal constitutively expressed mRNA control. After 25 cycles, PCR products were analysed using analytical agarose-ethidium bromide electrophoresis.

### *Genetic mapping of the* R-BPMV *gene*

A Chi-squared (χ2) test was used to evaluate the goodness of fit of observed and expected segregation ratios. The MAPMAKER software version 3.0 [43] was used to map the *R-BPMV* resistance gene on the integrated linkage map of common bean using the set of 142 markers [44] as described in Geffroy *et al.* [45]. Linkage groups were established with a LOD threshold of 3.0 and a maximum recombination fraction of 0.3. The position of *R-BPMV* on LG B2 was estimated with a LOD threshold of 2.0 based on the “try” function. Map distances were estimated by the Kosambi mapping function [46].

## Additional file

Additional file 1: Figure S1.
*Bean pod mottle virus* (BPMV)-induced expression of the *green fluorescent protein* (*GFP*) gene in roots after rub inoculation of one primary leaf with leaf sap. Roots of *P. vulgaris* cv. Black Valentine plants infected with mock buffer, BPMV empty vector (BPMV-0) and *GFP*-expressing vector (BPMV-GFP) were rinsed and photographed at 21 days post-inoculation (dpi) under natural light (top panel) and UV light (middle panel). Epifluorescence microscopy detection of GFP fluorescence in roots of common bean plants (bottom panel). Scale bars are 250 μm. **Figure S2.** Stability of *green fluorescent protein* (*GFP*)-expression in *P. vulgaris* cv. Black Valentine after four serial inoculations. BPMV-GFP inoculated plants of a fourth serial inoculation were photographed under UV light, at 21 days post-inoculation (dpi). **Figure S3.** *Bean pod mottle virus* (BPMV)-induced expression of the *green fluorescent protein* (*GFP*) gene in leaves of *P. vulgaris* cultivars. BPMV-GFP inoculated plants were photographed under UV light, at 7 days post inoculation (dpi) for inoculated leaves (IL), and at 21 dpi and 28 dpi for systemic leaves (SL). **Figure S4.** Alignement of the 327-bp fragment of the *Glycine max PDS* ortholog (*GmPDS*) with the corresponding regions of *PvPDS* from *P. vulgaris* cv. G19833 (*PvaPDS*) and BAT93 (*PvmPDS*). The longest region presenting 100% nucleic identity has a length of 52 bp and is indicated with light grey characters on the alignment. Overall identity of the three sequences on the 327 bp region is 92%.
