# Supplementary material for: The “one-step” Bean pod mottle virus (BPMV)-derived vector is a functional genomics tool for efficient overexpression of heterologous protein, virus-induced gene silencing and genetic mapping of BPMV R-gene in common bean (Phaseolus vulgaris L.)
Source: BMC Plant Biol. 2014 Aug 29;14:232. doi: 10.1186/s12870-014-0232-4 (PMC4163167; doi:10.1186/s12870-014-0232-4)
Supplement: Additional file 1: Figure S1. — Bean pod mottle virus (BPMV)-induced expression of the green fluorescent protein (GFP) gene in roots after rub inoculation of one primary leaf with leaf sap. Roots of P. vulgaris cv. Black Valentine plants infected with mock buffer, BPMV empty vector (BPMV-0) and GFP-expressing vector (BPMV-GFP) were rinsed and photographed at 21 days post-inoculation (dpi) under natural light (top panel) and UV light (middle panel). Epifluorescence microscopy detection of GFP fluorescence in roots of common bean plants (bottom panel). Scale bars are 250 μm. Figure S2. Stability of green fluorescent protein (GFP)-expression in P. vulgaris cv. Black Valentine after four serial inoculations. BPMV-GFP inoculated plants of a fourth serial inoculation were photographed under UV light, at 21 days post-inoculation (dpi). Figure S3. Bean pod mottle virus (BPMV)-induced expression of the green fluorescent protein (GFP) gene in leaves of P. vulgaris cultivars. BPMV-GFP inoculated plants were photographed under UV light, at 7 days post inoculation (dpi) for inoculated leaves (IL), and at 21 dpi and 28 dpi for systemic leaves (SL). Figure S4. Alignement of the 327-bp fragment of the Glycine max PDS ortholog (GmPDS) with the corresponding regions of PvPDS from P. vulgaris cv. G19833 (PvaPDS) and BAT93 (PvmPDS). The longest region presenting 100% nucleic identity has a length of 52 bp and is indicated with light grey characters on the alignment. Overall identity of the three sequences on the 327 bp region is 92%. [file 12870_2014_232_MOESM1_ESM.pptx]

## Slide 1
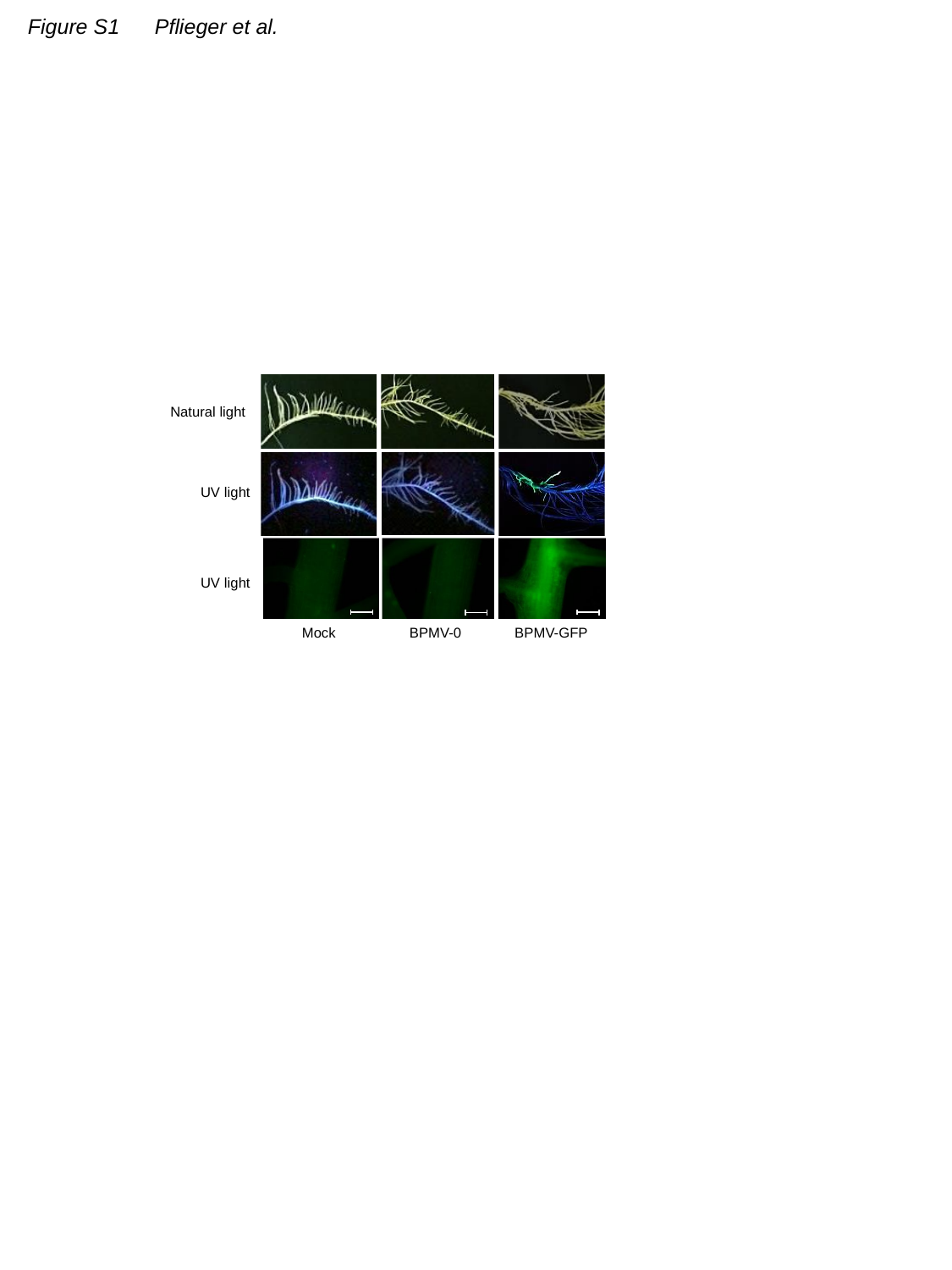

Figure S1	Pflieger et al.
Natural light
UV light
UV light
Mock
BPMV-0
BPMV-GFP

## Slide 2
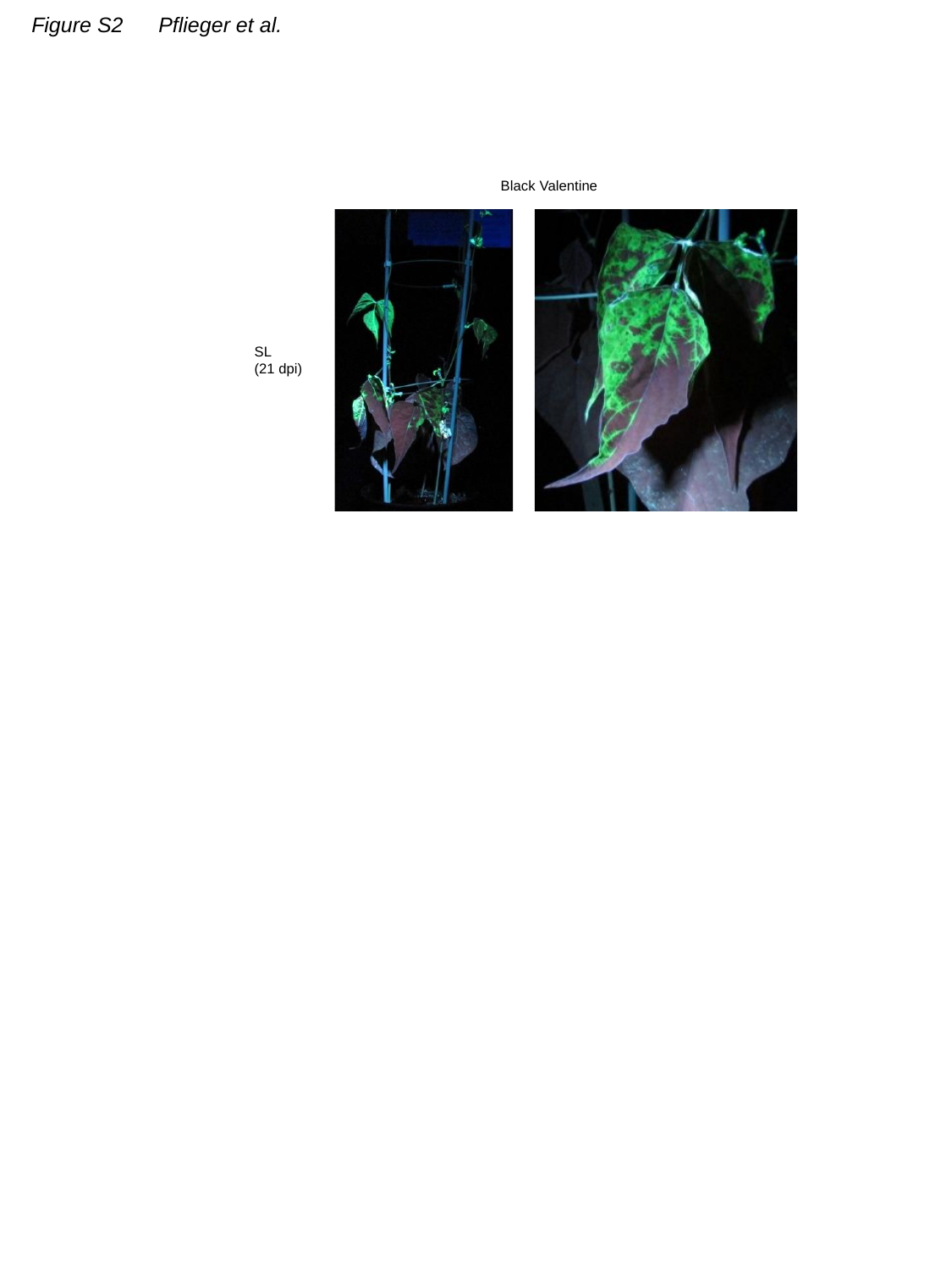

Figure S2	Pflieger et al.
Black Valentine
SL
(21 dpi)

## Slide 3
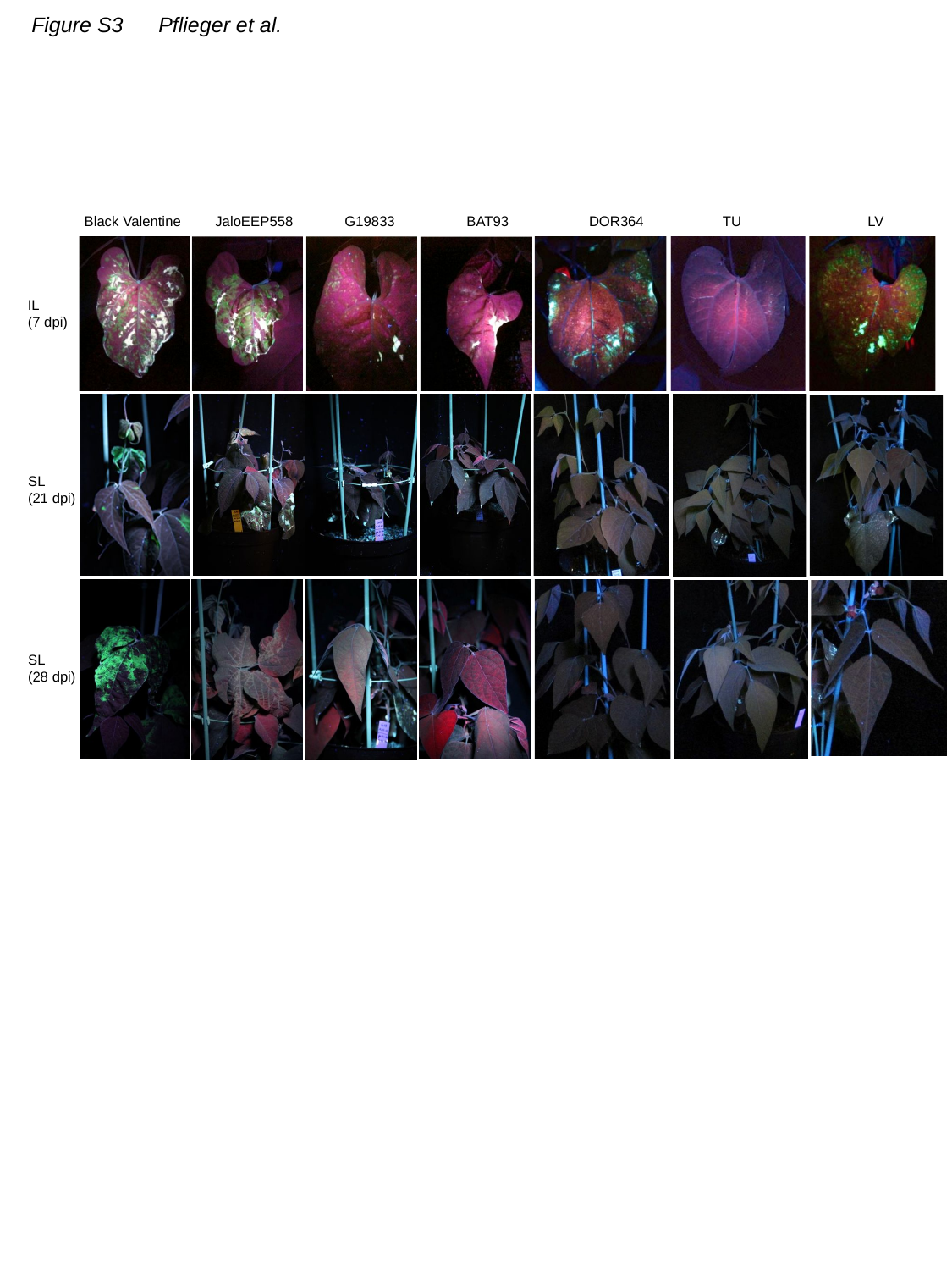

Figure S3	Pflieger et al.
Black Valentine
JaloEEP558
G19833
BAT93
DOR364
TU
LV
IL
(7 dpi)
SL
(21 dpi)
SL
(28 dpi)

## Slide 4
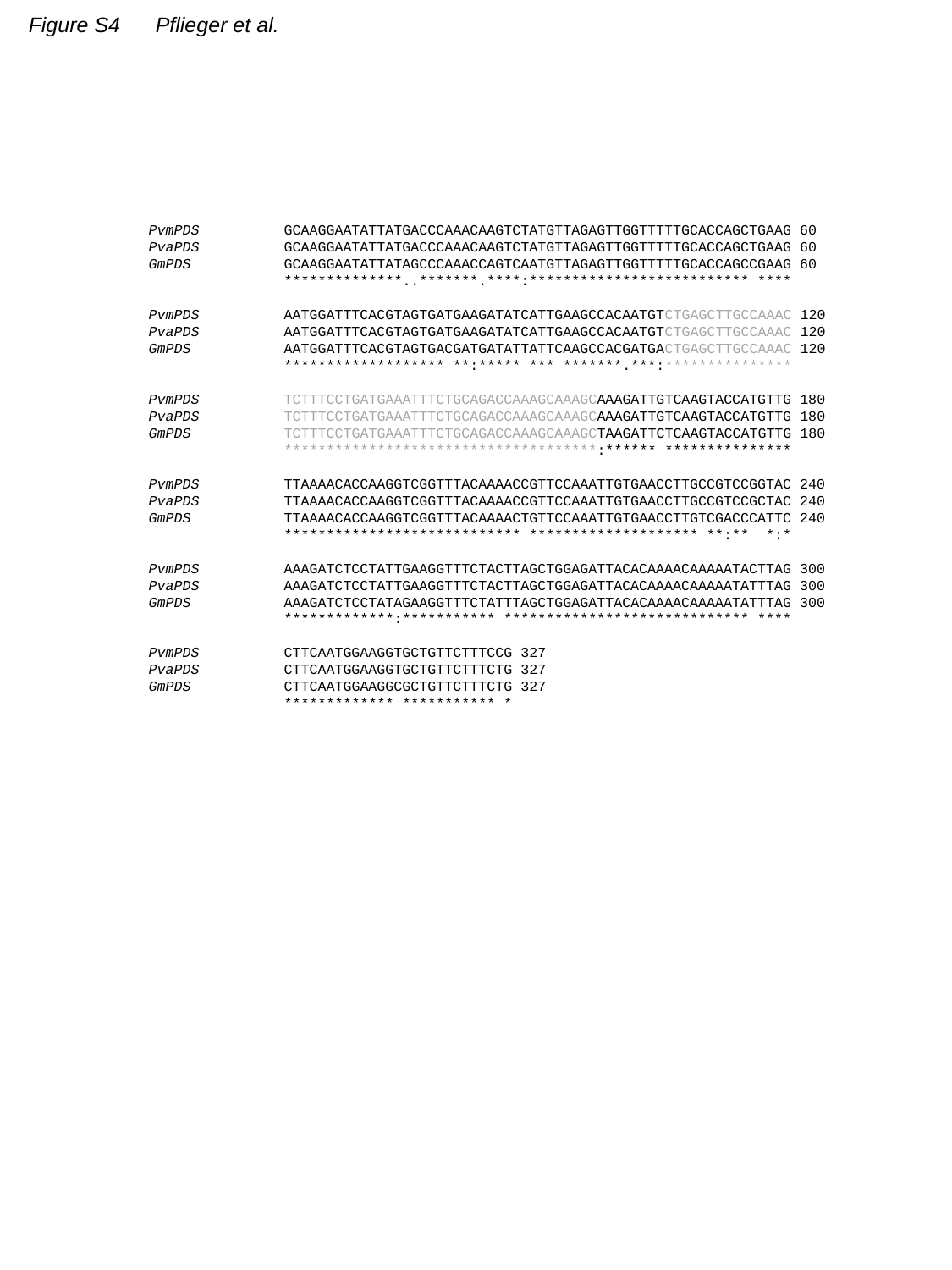

Figure S4	Pflieger et al.
PvmPDS GCAAGGAATATTATGACCCAAACAAGTCTATGTTAGAGTTGGTTTTTGCACCAGCTGAAG 60
PvaPDS GCAAGGAATATTATGACCCAAACAAGTCTATGTTAGAGTTGGTTTTTGCACCAGCTGAAG 60
GmPDS GCAAGGAATATTATAGCCCAAACCAGTCAATGTTAGAGTTGGTTTTTGCACCAGCCGAAG 60
 **************..*******.****:************************** ****
PvmPDS AATGGATTTCACGTAGTGATGAAGATATCATTGAAGCCACAATGTCTGAGCTTGCCAAAC 120
PvaPDS AATGGATTTCACGTAGTGATGAAGATATCATTGAAGCCACAATGTCTGAGCTTGCCAAAC 120
GmPDS AATGGATTTCACGTAGTGACGATGATATTATTCAAGCCACGATGACTGAGCTTGCCAAAC 120
 ******************* **:***** *** *******.***:***************
PvmPDS TCTTTCCTGATGAAATTTCTGCAGACCAAAGCAAAGCAAAGATTGTCAAGTACCATGTTG 180
PvaPDS TCTTTCCTGATGAAATTTCTGCAGACCAAAGCAAAGCAAAGATTGTCAAGTACCATGTTG 180
GmPDS TCTTTCCTGATGAAATTTCTGCAGACCAAAGCAAAGCTAAGATTCTCAAGTACCATGTTG 180
 *************************************:****** ***************
PvmPDS TTAAAACACCAAGGTCGGTTTACAAAACCGTTCCAAATTGTGAACCTTGCCGTCCGGTAC 240
PvaPDS TTAAAACACCAAGGTCGGTTTACAAAACCGTTCCAAATTGTGAACCTTGCCGTCCGCTAC 240
GmPDS TTAAAACACCAAGGTCGGTTTACAAAACTGTTCCAAATTGTGAACCTTGTCGACCCATTC 240
 **************************** ******************** **:** *:*
PvmPDS AAAGATCTCCTATTGAAGGTTTCTACTTAGCTGGAGATTACACAAAACAAAAATACTTAG 300
PvaPDS AAAGATCTCCTATTGAAGGTTTCTACTTAGCTGGAGATTACACAAAACAAAAATATTTAG 300
GmPDS AAAGATCTCCTATAGAAGGTTTCTATTTAGCTGGAGATTACACAAAACAAAAATATTTAG 300
 *************:*********** ***************************** ****
PvmPDS CTTCAATGGAAGGTGCTGTTCTTTCCG 327
PvaPDS CTTCAATGGAAGGTGCTGTTCTTTCTG 327
GmPDS CTTCAATGGAAGGCGCTGTTCTTTCTG 327
 ************* *********** *
